# Sol–Gel Approach for Fabricating Silica/Epoxy Nanocomposites

**DOI:** 10.3390/polym15142987

**Published:** 2023-07-08

**Authors:** Francesco Branda, Rossella Grappa, Aniello Costantini, Giuseppina Luciani

**Affiliations:** Department of Chemical Materials and Industrial Production Engineering (DICMaPI), University of Naples Federico II, P.le Tecchio 80, 80125 Naples, Italy; rossella.grappa@unina.it (R.G.); aniello.costantini@unina.it (A.C.); giuseppina.luciani@unina.it (G.L.)

**Keywords:** sol–gel, silica in situ formation mechanism, highly engineered nanoparticles, nanocomposites, epoxy, flame retardancy

## Abstract

This review focuses on the opportunities provided by sol–gel chemistry for the production of silica/epoxy nanocomposites, with significant representative examples of the “extra situ” approach and an updated description of the “in situ” strategy. The “extra situ” strategy enables the creation of nanocomposites containing highly engineered nanoparticles. The “in situ” approach is a very promising synthesis route that allows us to produce, in a much easier and eco−friendly manner, properly flame−retarded silica/epoxy nanocomposites endowed with very interesting properties. The review highlights the recently proposed mechanism of nanoparticles formation, which is expected to help to design the synthesis strategies of nanocomposites, changing their composition (both for the nanoparticle and matrix nature) and with in situ−generated nanoparticles possibly more complex than the ones obtained, until today, through this route.

## 1. Introduction

Epoxy resins, thanks to their many good properties, including high Young’s modulus and tensile strength, solvent resistance, thermal stability and good thermal insulation, have found relevant industrial applications in the fields of adhesives, laminates, electronic devices, coatings, and aerospace [1,2]. Recently, the focus was enlarged to include novel multifunctional epoxy nanocomposites with unique anticorrosive, optical, electrical and magnetic properties [1,2].

Notably, great interest is paid to the silica/epoxy nanocomposites, which remain among the most investigated systems, as confirmed by the large number of studies published in the last few years [1,2].

Silica improves the electrical insulating properties of epoxies [3], particularly AC dielectric breakdown strength [4,5], which is of paramount importance in the applications of epoxy as polymer-based dielectric.

Furthermore, silica is one of the most studied fillers added to modified epoxy polymer networks to improve mechanical properties (elastic modulus, hardness, impact resistance and fracture toughness) for applications in the advanced sector of industries such as aerospace, automotive and locomotive, wind energy and defense [6,7]. In the case of fracture toughness, a typical “nano-effect” was documented [6]. The influence of a silane coupling agent on the structure and thermomechanical properties of silica epoxy nanocomposites was investigated through molecular dynamics simulation [8]. The proper selection of the silane coupling agent for the modification of filler surface modification enhanced their thermomechanical properties [8]. Silica nanoparticles functionalized with (3-glycidyloxypropyl) trimethoxysilane (GPTMS) were proven to be an excellent cost-effective additive to mitigate the brittle failure in epoxy composites [7]. The effect of interfacial interaction on the thermal properties of silica/epoxy nanocomposites and the rheological properties of the liquid-epoxy-based nanosuspensions were also investigated [9]. The establishment of attractive interfacial interaction reduced the viscosity and improved viscosity stability and the thermal stability of epoxy nanocomposites.

Silica nanoparticles are one of the most important nanofillers introduced in the so-called fiber-reinforced epoxy nanocomposite (FRENC), that is, fiber-reinforced polymer composite (FRPC), in which nanofillers are added to the epoxy matrix to improve the fracture energy of the cured epoxy [10].

Self-healing epoxy-based nanocomposites containing organosilane-modified silica nanoparticles and self-healing microcapsules were successfully prepared to improve an epoxy coating anticorrosion performance [11]. Nanosilica addition to epoxy had a plasticizing effect and improved the resistance to erosion by cavitation [12].

There are two challenges in the production of good nanocomposites: achieving a fine dispersion of nanoparticles, and achieving well-tailored nanofiller/polymer interactions. Both targets require the synthesis of functionalized nanoparticles [1,13]. Sol–gel chemistry is a useful route to meet the scope. It allows us to synthesize highly sophisticated engineered nanoparticles [2,14,15,16,17,18,19,20,21,22] to be dispersed into the polymeric matrix. The in situ sol–gel process that allows “solvent-free one-pot’’ strategies with great economical advantages and a minimum environmental impact is also captivating [23].

Very recently [24,25,26,27,28,29], it was also demonstrated that the in situ strategy allows us to easily obtain flame-retarded silica epoxy nanocomposites, even with UL90-V0 rating, that has self-extinguishing fire behavior. This is a relevant result. Very recently, it was reported that the cure of a new bio-based epoxy monomer of diglycidyl ether luteolin (DGEL) with 4, 4′-diaminodiphenyl sulfone (DDS) resulted in a polymer that passed the V-0 rating in UL-94 tests [30]. Generally speaking, however, the exploitation of known epoxy-based materials requires the proper addition of flame retardants to pass V-0 rating in UL-94 tests. Moreover, new findings from the use of high-resolution transmission electronic microscopy (HRTEM) and a combined small and wide-angle X-ray scattering (SAXS) by means of a multirange Ganesha 300 XL+ device over an unprecedented q range (0.02–25 nm^−1^) demonstrated an unexpected multisheet structure of the nanosilica [31,32,33]. In view of the recent advances in the design, fabrication and theoretical achievements in silica–epoxy nanocomposites, this review is devoted to highlighting the opportunities offered by sol–gel chemistry with significative addressing examples and an updated description of the developments of the in situ strategies to make silica–epoxy nanocomposites. The focus is on the epoxy/silica system, but will include the developments of the in situ strategy that may be easily extended to other compositions [33].

## 2. The Sol–Gel Route—A Few Basic Concepts

The sol–gel process is an outstanding route for both the synthesis of nanoparticles and their functionalization [2,14,20,21]. It dates back to the beginning of the XX century. In 1926, Freundlich [34] reported that silicic acid could be obtained from the hydrolysis of tetraethoxysilane in excess water:

Si(OC_2_H_5_)_4_ + 2 H_2_O → Si(OH)_4_ + 4 C_2_H_5_OH (1)

He reported, however, that the diffusion rate of the obtained acid solution through parchments or animal membranes slowly reduced with time, while solution thickening until gelling was observed. These are reported [14] to be the first evidences of the so-called “sol–gel process” based on the strong tendency of the formed silicic acid to condensate, resulting in products of much greater molecular weight.

When the precursor is a silicon alkoxide, taking also into account that hydrolysis and polycondensation reactions occur simultaneously, the basic scheme of the sol–gel chemistry can be better written as follows:

Si(OR)_4_ + *n*H_2_O → Si(OR)_4−*n*_(OH)*_n_* + *n*ROH (2)

**≡**Si-OH + HO-Si**≡** → **≡**Si-O-Si**≡** + H_2_O (3)

**≡**Si-OH + RO-Si**≡** → **≡**Si-O-Si**≡** + ROH (4)

Polycondensation (Reactions (3) and (4)) turns monomers into oligomers and, finally, into inorganic polymers in the form of gels.

The gels may be easily converted into glasses or ceramics at very advantageous temperatures. It is worth reminding readers about the paper dated 1971, entitled “New routes to multicomponent oxide glasses”, in which Dislich [35] described a method to produce a Pyrex-type glass (in the form of relatively big samples measuring 4 cm × 1.5 cm × 2 mm) at a surprisingly low temperature [36] through hot-pressing gel crumbs obtained from solutions of alkoxides of sodium, boron and aluminum at 630 °C. The temperature was much lower than 1500 °C required by conventional glass-making techniques. However, by properly selecting the experimental conditions, films, fibers and particles are easily obtained [14,36,37,38].

### Functionalized Nanoparticles through Sol–Gel Chemistry

**Figure 1:**
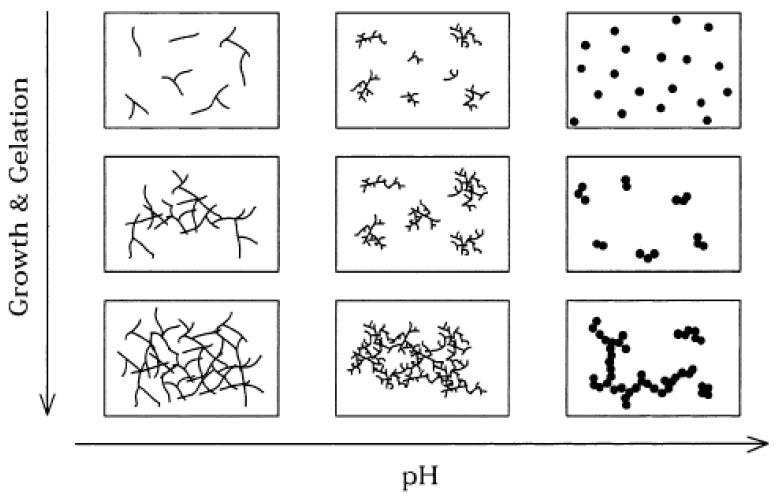
Effects of pH on particle morphology in sol–gel reactions. At low pH, a polymer gel forms, while a particulate gel forms at high pH. Reprinted with permission from [39]. Copyright 2023 Swiss Chemical Society.

It is recognized that the Reactions (2)–(4) have a nucleophilic substitution mechanism [14,20,21,39]. As a consequence, the resulting gel structure depends on whether the reactions are acid or base catalyzed. In an acid environment, initial silicatic chains formation is subsequently followed by branching and cross-linking [14,20,21,39]. Under basic conditions, the gel is the result of the initially formed cross-linking of large particulate agglomerates (Figure 1) [14,20,21,39].

The latter mechanism is exploited in the Stober method [38,40]. The method allows us to easily obtain monodisperse systems of spherical silica gel particles from hydro-alcoholic solutions of silicon alkoxides (typically TEOS: tetraethoxysilane) in the presence of ammonia. By changing the water and ammonia content, the particle size can be tuned from a few nanometers to the micron size. Unfortunately, the yield is low: the solid content achieves relatively low amounts (i.e., 3% at a TEOS content of 0.5 M). This indicates the need for high solvent quantities (the EtOH/TEOS weight ratio may be close to 10). However, the synthesis occurs at room temperature. These mild synthesis conditions allow the in situ formation of the inorganic phase in the presence of organic moieties like surfactants, monomers or polymers. Such features are exploited in the synthesis of mesoporous glasses or particles possessing very high specific surface areas (as high as 1000 m^2^/g) with pore sizes in the range 2–50 nm [41,42,43,44,45]. Their synthesis exploits the templating action of surfactant molecules during the sol–gel synthesis [45,46,47,48,49,50,51]. Very great interest was also raised by the discovery of mesoporous nanoparticles possessing a surprising wrinkled aspect [45,52]. They are obtained from oil-in-water macroemulsions, wherein droplets of bicontinuous microemulsions are dispersed. All reactions occur in the droplets. The mechanism is based on the hypothesis that TEOS dissolved in the oil layer comes into contact with water at the emulsion interface where hydrolysis and condensation reactions occur, finally resulting in wrinkled nanoparticles [52].

However, as reminded in the Introduction section, the nanoparticle dispersion into a polymer matrix requires their functionalization [1,53]. Indeed, in the absence of interactions between polymer and matrix, the interface energy of polymer and particles per unit volume of nanocomposite, E_ν_, has the maximum value, E_νmax_:

E_ν,max_ = S_v_ (γ_i_ + γ_p_) (5)

where S_v_ is the interface area per unit volume and, γ_i_ and γ_p_ are the surface energy of inorganic particles and polymer, respectively. The specific surface area of the particles in a nanocomposite is, of course, very high, leading to very high values of E_ν,max._ An obvious way the system has to reduce E_ν,max_ is through agglomeration, which causes S_v_ to decrease. The tendency to agglomerate may be eliminated or reduced by promoting interactions between particles and polymer matrix or an encapsulation of the particles in a shell of strongly adhering organic compounds [13]. In this last case, in fact, the two opposite surfaces both have an organic nature, assuring a lower E_ν,max_ with respect to the inorganic/organic case. In fact, the surface energy of inorganic (0.5–2.5 J/m^2^) is much higher than the organic one (0.01–0.05 J/m^2^). The problem of the nanoparticles dispersion is therefore strictly related to the tailoring of the interface that by itself strongly affects the final properties of the composite [13,22,53]. Many routes may be followed; wherein much of which, the sol–gel technique plays a central role. The topic was cleverly reviewed by Bourgeat-Lamie with numerous and somewhat exhaustive descriptions of examples [22].

**Scheme 1:**
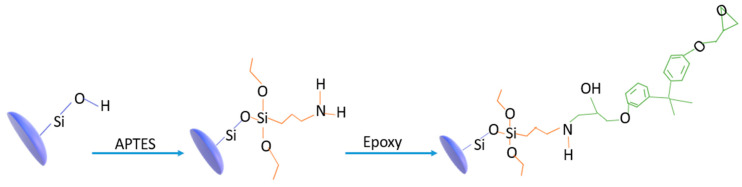
Functionalization through coupling agent. APTES anchors to the silica surface thanks to condensation reaction with silicatic surface silanols. The reaction of amino group with oxirane of epoxy allows, finally, a strong covalent bond matrix/silcatic surface to be set up.

The sol–gel chemistry provides easy and effective ways to functionalize the silica nanoparticles through the use of the so many available silane coupling agents [54,55]. These are usually silane of the formula:

R − (CH_2_)n − Si− X_3_ (6)

where X is a hydrolizable group (commonly an alkoxide or halogen moiety) and R is an organofunctional group. Common examples for epoxy matrix are: 3-aminopropyltriethoxysilane (APTES) and 3-glycidoxypropyltrimethoxysilane (GPTMS). As can be seen, the coupling agent must have at least two different functional groups. One of them (ethoxy in the case of APTES and methoxy in the case of GPTMS) assures a stable bond with the silica nanoparticle substrate through condensation with silanols of silica (Reaction (4)), as described in Scheme 1. Alternatively, the condensation may directly involve an ethoxy group (see Reaction (4)). The other functional group (amino group for APTES and oxirane group for GPTMS), generally linked to silicon through a trimethylene group, survives the sol–gel chemistry and allows the formation of strong covalent bonds with the hosting matrix, thanks to the reaction with the functional group of the matrix (i.e., reaction between amino group of APTES and oxirane ring of epoxy resin). This is described in Scheme 1.

However, depending on the application of interest, different forms of functionalization have been described in the literature that find in the sol–gel silica a valid substrate thanks to the recognized high reactivity of sol–gel particles due to the high silanol groups’ surface density and gel structure [22]. Highly engineered nanoparticles were prepared as described in the following.

## 3. Nanocomposite Synthesis

It is useful to distinguish two approaches in the use of the fabrication route from sol–gel to nanocomposite:

- Extra situ: It consists in making the nanoparticles and disperse them in the matrix.
- In situ: It exploits the “mildness” of the reaction conditions that allows us to prepare nanoparticles in the presence of organic moieties like polymers or monomers.

### 3.1. Synthesis by Dispersion of Preformed Nanoparticles (Ex Situ Process)

**Figure 2:**
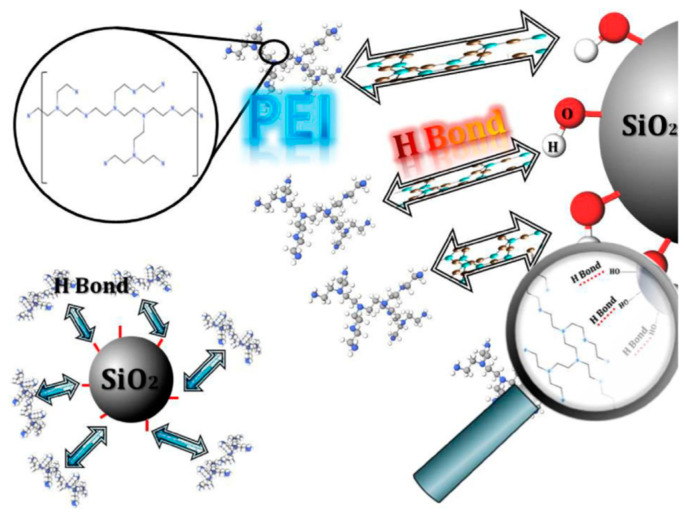
Surface modification mechanism of silica particles by PEI, showing H-bonds formed between silanol groups of silica and N–H groups of polyethyleneimine. Reprinted with permission from [56]. Copyright 2023 Elsevier.

There are many examples in the literature. The nanoparticles can be dispersed after a compatibilization through a coupling agent, as described previously. Alternatively, complex nanostructures can be ex situ-formed, starting from the sol–gel nanoparticles to the various processes well-described in the literature. We will mention a few representative examples. Ghiyasi et al. decorated Stober particles with polyethyleneimine through H bonds and impregnated them with an epoxy/amine system to produce nanostructured coatings (as described in Figure 2) [56].

**Figure 3:**
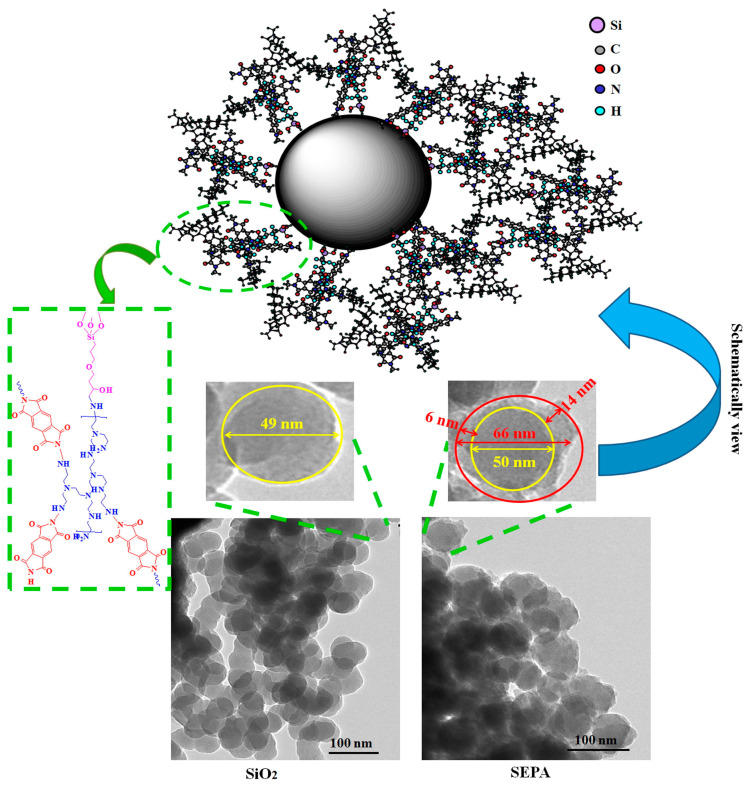
TEM micrographs of SiO_2_ and SEPA clearly showing the formation of a layer 6–14 nm thick. Reprinted with permission from [57]. Copyright 2023 Elsevier.

Jouyandeh [57] similarly decorated Stober particles obtaining a stronger covalent functionalization. In fact, the amine groups of polyethyleneimine (PEI) were preventively reacted with the epoxide one of [3-(2,3-epoxypropoxy) propyl] trimethoxysilane (EPPTMS). First of all, an adduct was formed thanks to a reaction of the amine groups of polyethyleneimine (PEI) with the oxirane ring of [3-(2,3-epoxypropoxy) propyl] trimethoxysilane (EPPTMS). “Super-reactive nitrogen-rich surface-decorated” SiO_2_ nanoparticles (SEPA) were therefore obtained thanks to the reaction of the silanol groups present on Stober particles with the methoxy groups of the hybrid molecule (similarly to that which was described in the previous paragraph for grafting APTES). A TEM image is shown in Figure 3.

The dispersion in epoxy resin caused, finally, a significant increase in the glass transition temperature of the cured nanocomposite: at a concentration as low as 0.5 wt.%, the glass transition temperature of epoxy increased from 128 to 156 °C.

**Figure 4:**
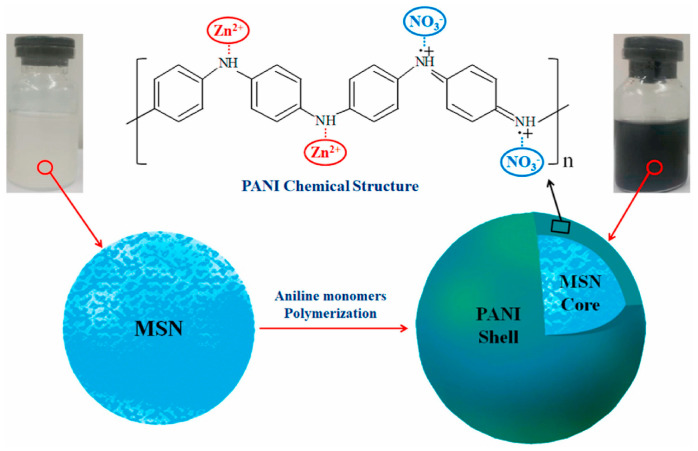
Decoration process of nanoparticle depicting the formation of a PANI shell around the mesoporous silica nanoparticle (MSN). Reprinted with permission from [58]. Copyright 2023 Elsevier.

Haddadi [58] prepared epoxy nanocomposite coatings containing zinc-doped silica/polyaniline core/shell nanoparticles. Mesoporous silica nanoparticles were first prepared with the above-described sol–gel chemistry in the presence of surfactants. The nanoparticles were successively decorated with positively charged polyaniline layers by the in situ polymerization of aniline monomers in the presence of zinc cations. The decorated particles were finally dispersed in the epoxy resin, as shown in Figure 4.

**Figure 5:**
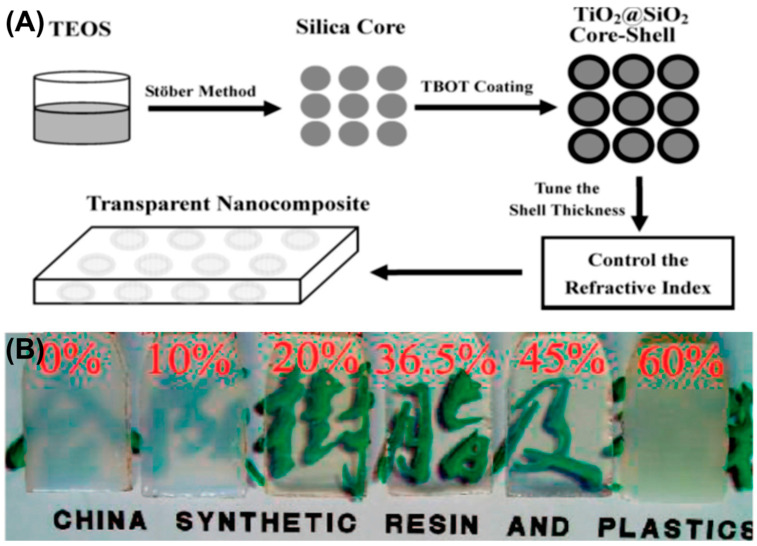
(**A**) Silica/titania core/shell nanoparticles scheme; (**B**) photo of nanocomposites as a function of the shell weight percentage. Reprinted with permission from [59]. Copyright 2023 American Chemical Society.

Li et al. [59] proposed an easy way to prepare a “highly” transparent polymer nanocomposite through the addition of core/shell nanoparticles projected to have a refractive index very close to the matrix one. Silica/titania core/shell nanoparticles were obtained through the hydrolysis of tetrabutyl orthotitanate (TBOT) continuously fed to a colloidal solution of silica Stober nanoparticles, as described in Figure 5. The highest transparency is observed at the 36.5 wt.% titania shell content.

Picu [60] studied the effect of tunable filler/matrix interface on the toughening of nanosilica reinforced epoxy. He produced epoxy nanocomposites reinforced with Stober silica nanoparticles, which were decorated with photosensitive phenylazide moieties. In more detail, the successive reaction of 4-azidobenzoic acid with oxalyl chloride ((COCl)_2_) and APTES resulted in a phenylazide–silane adduct possessing at one end a silicon bearing three ethoxy groups that allowed for anchoring to Stober silica nanoparticles through the well-known condensation reactions (Reactions (3) and (4)). The photosensitive phenylazide moieties allowed, by exposure to UV radiation, us to control the interface mechanical properties. The above-reported examples are only a few of the various applications of sol–gel chemistry. As can be seen, sol–gel chemistry allows us to build up highly engineered nanoparticles tailored to the specific applications.

### 3.2. In Situ Sol–Gel Chemistry

**Scheme 2:**
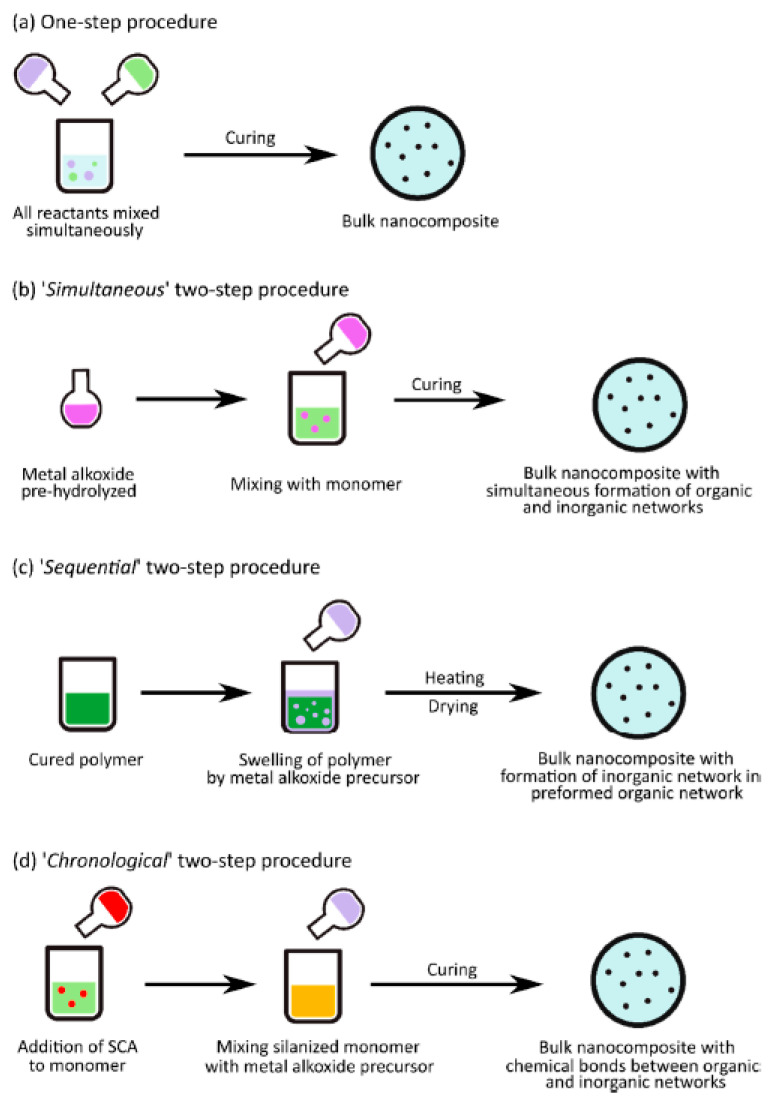
Scheme of (**a**) one-step procedure, (**b**) simultaneous, (**c**) sequential, and (**d**) chronological two-step procedure. The difference is in the order of addition of reagents. Reprinted with permission from [23]. Copyright 2023 MDPI.

The above-described ex situ route, that is synthesis by the dispersion of preformed nanoparticles, allows us to obtain interesting complex functional nanomaterials at the expense of the use of large quantities of solvents, which are often very harmful. Moreover, the synthesis requires several successive processing steps for the nanoparticles synthesis, their functionalization, multiple washing and solvent removal operations. For these reasons, starting from the pioneering works by Mark et al. [61] and Ning et al. [62] many papers have been published describing successful in situ strategies, which were recently well reviewed [23]. In situ approaches exploit the mildness of sol–gel reactions to prepare nanoparticles in the presence of organic moieties, including polymers or monomers. Mark et al. [61] envisaged a number of potential practical advantages of filling a network in such a novel manner: the avoidance of filler blending and other difficult processing techniques; the achievement of particle sizes not otherwise attainable; the control of particle size distribution and agglomeration; and the tuning of the nature of the particle surface and reinforcement of some shapes (for example, thin films) that are difficult to process if already filled. In the in situ processes, nanoparticle formation and the cure of epoxy resin occur, simultaneously or successively, in the same pot, as represented in Scheme 2. The procedure differences arise from the order in which the reagents are added. It is worth reminding that, generally speaking, the nanocomposite formation requires the presence of the epoxy resin with its curing agent together with the reagents of the sol–gel synthesis of functionalized inorganic particles: the inorganic precursor (usually a metal alkoxide like TEOS), water, alcohol and a coupling agent.

Four strategies may be envisaged [23], as schematically shown in Scheme 2:

- A “one-step” procedure: All the reagents are mixed simultaneously.
- A “simultaneous two-step” procedure: The silica precursor is first pre-hydrolyzed in a first step, then the monomer and the curing agent are added in order to build up both organic and inorganic networks.
- A “sequential two-step” procedure: The polymer is cured; successive swelling allows the reagent mixture of inorganic precursor to diffuse into its pores where the inorganic network is built up.
- A “chronological two-step” procedure: A mixture of epoxy resin and a coupling agent is left to react and produce a hybrid molecule; the successive addition of the remaining reagents of the inorganic precursor mixture and the curing agent finally leads to the nanocomposite.

Matejka et al. [23,63,64,65] explored and compared the first three routes for the synthesis of silica/epoxy nanocomposites. They found that the synthesis route had a strong influence on the nanocomposite structure. The nanocomposites were determined to have a “bicontinuous morphology”, in agreement with previous findings [66], which recognized that an “interpenetrating polymer network” was obtained. Piscitelli et al. [67,68] successfully exploited the “simultaneous two-step” route to produce nanocomposites with an improved resistance to plastic deformation. Their coupling agent was GPTMS (γ-glycidoxypropyl-trimethoxysilane), sometimes indicated also as GLYMO or GOTMS. On the basis of Si NMR, TEM, SAXS and WAXS results, they proposed the presence of two co-continuous phases. In the previous paper [67], they hypothesized the “presence of both plasticizing flexible linear siloxane sequences and reinforcing nanosized silica particles and branched silsesquioxanes (SSQO) structures”. Afzal et al. [69] found that higher decomposition temperatures are recorded when using the coupling agent GPTES (γ-glycidoxypropyltriethoxysilane).

The “simultaneous two-step” route was also exploited by Wu et al. [70] to prepare an interesting epoxy/inorganic hybrid that resists for moderate-temperature and low-pressure imprint lithography using GPTMS as coupling agent. In this case, the pre-hydrolysis step was performed in the presence of the epoxy. The “chronological two-step” procedure was applied by many authors employing different coupling agents: IPTS (3-isocyanatopropyltriethoxysilane) [71,72,73], APTMS ((3-aminopropyl) trimethoxysilane) [74], APTES (3-aminopropyl-triethoxysilane) [24,25,26,27,28,29,31,32,33,75,76,77,78,79], GPTMS (γ-glycidoxypropyl-trimethoxysilane)—sometimes indicated also as GLYMO or GOTMS [78,80]. Improvements of mechanical properties [72,73,74,76], glass transition temperature, storage modulus [75] and better high voltage insulation properties with respect to the neat epoxy matrixes [77,78,79] were recorded.

More interestingly, it is worth reminding that the in situ strategies, particularly the “chronological two-step” approach, allows for easy modifications to be finalized for the improvement of the flame retardancy-to-self-extinguishing behavior. Chiang et al. [71] showed that using a phosphorus-containing silica precursor, like diethylphosphatoethyltriethoxysilane, the fire behavior was improved. In fact, the limiting oxygen index (LOI) (ASTM D 2836), defined as the minimum percentage of O_2_ in a mixture of O_2_ and N_2_ supporting flaming combustion, increased from 24 (for the neat polymer) to 32 (for the nanocomposite). Very interesting results were also obtained by introducing other reagents in the pot, as will be described in a successive paragraph, finally reaching a UL94-V0 rating that is self-extinguishing fire behavior. An intriguing procedure that can be associated with the “chronological two-step” process was followed by Mascia et al. [81,82] and Prezzi et al. [83]. The key feature is that epoxy was initially mixed with APTES in a xylene or xylene/butanol solution and subsequently mixed with a pre-polymerized alkoxysilane solution (TEOS, GOTMS, water ethanol catalyst). The scope was, however, the same pursued as in other works: to obtain a hybrid molecule thanks to the reaction of an amino group of APTES with the oxirane of epoxy. GOTMS was also used in the pre-polymerized alkoxysilane solution. The nanocomposite barrier properties were studied and a large reduction in the equilibrium absorption of aprotic solvents, including THF, was reported. They also found a small reduction in the absorption of protic solvents, such as CH_3_OH. The formation of bicontinuous-phase nanocomposites was well-proven through TEM and XRD measurements. Improvements of barrier properties are linked to this kind of structure [82]. The extent depends on the type of alkoxysilane used for the functionalization of the resin. On the other hand, particulate nanocomposites provide only marginal improvements in solvent resistance [82]. In another paper [83], the resin was initially grafted with 3-isocyanatopropyltriethoxysilane (IPTS o ICTES), mercapto γ-propyltrimethoxysilane (MPTMS) or APTES in xylene/butanol solution; another difference is that components of the precursor solution were pre-reacted after mixing with epoxy-silanized solution. A denser network with a higher glass transition temperature than the neat resin was formed in the case of coupling agents with a basic character (amine silane type).

## 4. Syntheses That Progress beyond the Classical In Situ Batch Composition

**Figure 6:**
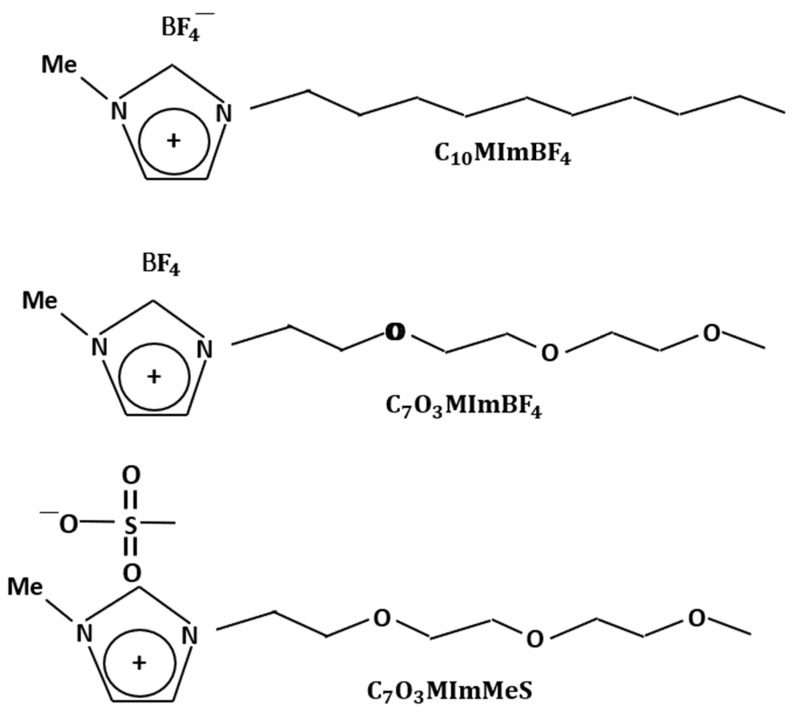
Structure of ionic liquids.

The classical batch of the in situ process contains the reagents that are strictly necessary to produce the two phases: the resin with its curing agent, the inorganic precursor (usually a metal alcoxide like TEOS), water, alcohol and a coupling agent. However, the process can be modified by including other reagents or compounds. Modifications have been reported involving the use of ionic liquids, flame retardants and also biomasses like humic acid. These processes are described below. Alternative interesting procedures were developed [84,85,86] using room-temperature ionic liquids (IL), including 1-decyl-3-methylimidazolium tetrafluoroborate (C_10_MImBF_4_), 1-triethylene glycol monomethyl ether-3-methylimidazolium tetrafluoroborate (C_7_O_3_MImBF_4_) and 1-triethylene glycol monomethyl ether-3-methylimidazolium methanesulfonate (C_7_O_3_MImMeS), whose structures are shown in Figure 6.

**Figure 7:**
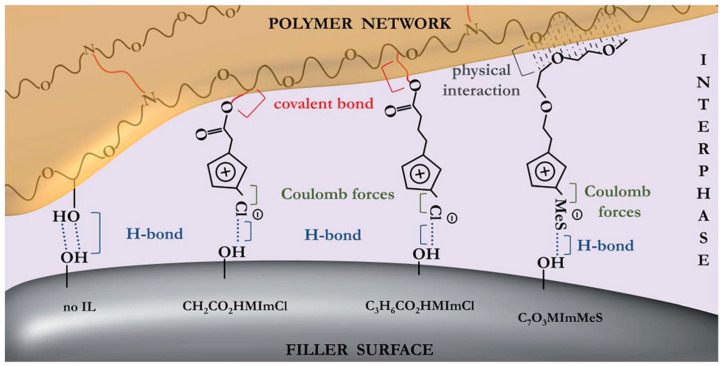
Representation of interaction between polymer network and filler surface beyond physical interactions; covalent bonds form thanks to reaction of carboxylic-ILs at the epoxy interface. Reprinted with permission from [86]. Copyright 2023 Royal Society of Chemistry.

IL are organic salts that are liquid below 100 °C. They have already been used as templates in the synthesis of silica mesoporous nanoparticles. Silica/epoxy nanocomposites were obtained through both the “one-step” and “simultaneous two-step” procedures. In the second case, the pre-hydrolized solution batch contained TEOS and IL in isopropyl alcohol solution, water and HCl as the catalyst. No silane coupling agent was used. Hybrids with diverse morphologies and improved mechanical properties were obtained depending on the chemical nature of IL that appeared to significantly influence silica structure and interphase interactions. Very fine hybrid morphology was obtained with 1-decyl-3-methylimidazolium tetrafluoroborate ionic liquid with well-dispersed silica nanodomains and a significative increase in rubbery modulus [84]. Successively, they studied the effect of adding to the pre-hydrolysis batch a silane coupling agent (3-glycidyloxypropyltrimethoxysilane (GPTMS)). They proved that the chemical interfacial bonding was improved with toughness increase without any significant loss in stiffness. C_7_O_3_MImMeS and GPTMS assured the best tensile property balance: the rubbery nanocomposite had a 6-times higher modulus and tensile strength as well as more than a 10-times higher energy to break the unmodified silica/epoxy nanocomposite [85]. Very interesting results were obtained when carboxylic-functionalized task-specific imidazolium ionic liquids (carboxylic-ILs) were used [86]. It was hypothesized that the reaction of carboxylic-ILs with epoxy created in situ a chemical bond as described in Figure 7 and allowed us to tune the filler/matrix interphase as described in the same figure.

The functionalized IL was introduced through both hydrolytic and non-hydrolytic processes that can be associated with the “simultaneous two-step” and “one-step” methods, respectively. Well-dispersed silica nanodomains were obtained with a morphology depending on structural modifications in the carboxylic-ILs. Rubbery and glassy epoxies’ toughness increased, respectively, of more than 7 times and almost twice in the case of the application of IL 1-carboxypropyl-3-methylimidazolium chloride, with respect to IL-free equivalents [86].

More recently, the “in situ” synthesis was also proven to be a promising route to obtain nanocomposites that satisfy the severe fire safety regulations whose satisfaction is a “sine qua non” prerequisite for almost all their applications. In this regard, it is worth reminding that many authors evidenced an increase in the thermal stability of in situ silica/epoxy nanocomposites with respect to the neat epoxy type [73,75,77]. Notably, the in situ strategies, particularly the “chronological two-step“ one, allows for easy modifications to be finalized for the improvement of flame retardancy-to-self-extinguishing behavior. Chiang et al. [71] showed that the use of a phosphorus-containing silica precursor, like diethylphosphatoethyltriethoxysilane, improved the fire behavior. Indeed, the limiting oxygen index (LOI) (ASTM D 2836), defined as the minimum percentage of O_2_ in a mixture of O_2_ and N_2_ supporting flaming combustion, increased from 24 (for neat polymer) to 32 (for nanocomposite).

**Figure 8:**
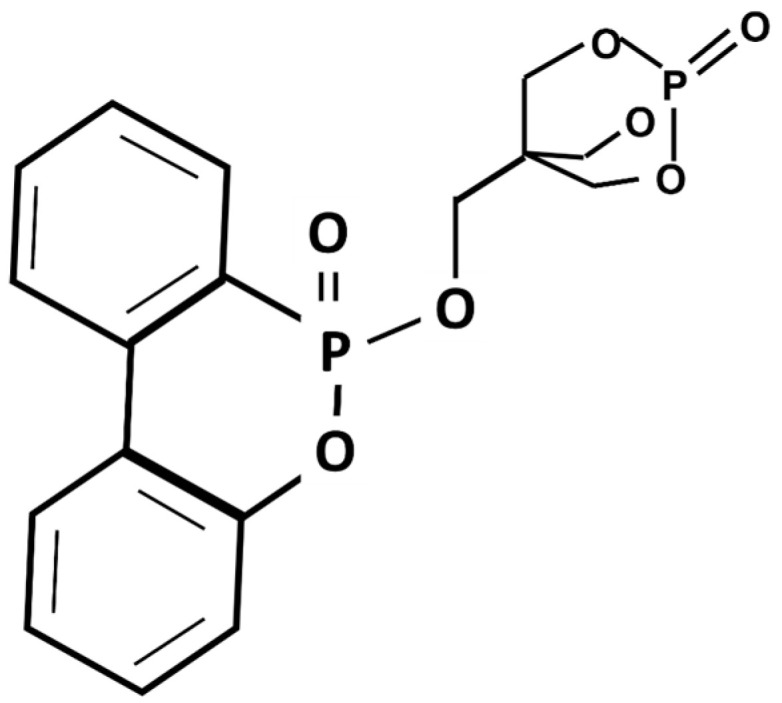
Chemical structure of 6H-dibenz [c, e] [1,2] oxaphosphorin,6-[(1-oxido-2,6,7-trioxa-1-phosphabicyclo [2.2.2] oct-4-yl) methoxy]-, 6-oxide (DP).

**Figure 9:**
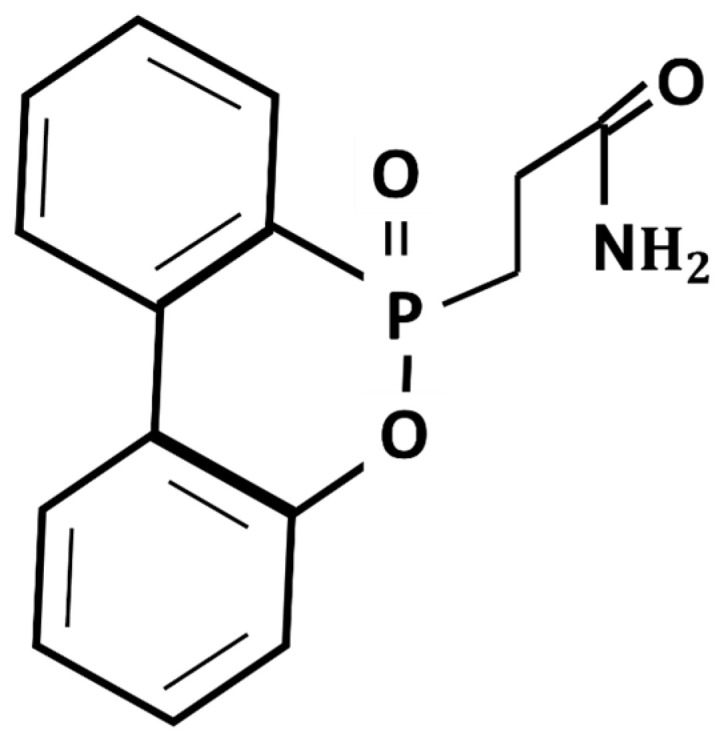
3-(6-oxidodibenzo [c, e] [1,2] oxaphosphinin-6-yl) propenamide (DA).

Bifulco et al. [24,25,26,27,28] and Venezia et al. [29] proved that flame retardants can be added in the course of the second step of the “chronological two-step” process with great impact on the fire behavior. Indeed, it is worth to point out that, also in the absence of traditional flame retardants, the in situ formation of nanosilica improves fire behavior. In particular, nanosilica prevented melt dripping in the course of vertical flame spread tests [24]. A remarkable decrease in the heat release rate (HRR) (about 40%), even at very low silica loadings (i.e., 2 wt.%), was recorded with cone calorimeter tests [24]. Similar results were obtained [27] by applying the “chronological two-step” procedure to the synthesis of biobased epoxy/silica nanocomposites, simply using the bioderived epoxy resin 2,5-bis[(oxyran-2-ylmethoxy) methyl] furan (BOMF) and methyl nadic anhydride as the curing agent. Silica/epoxy nanocomposites cured with cycloaliphatic hardener, with improved flame retardancy, were obtained by simply adding, in the “chronological two-step” procedure, phosphoric acid hydroalcoholic solution directly before the curing agent [28]. The self-extinguishing behavior of epoxy cured with a cycloaliphatic hardener was obtained by simply adding proper mixtures of traditional flame retardants in the course of the second step of the synthesis, as reported in the following [25,26]:

(a) A DOPO derivative phosphorus (P) flame-retardant, i.e., 6H-dibenz [c, e] [1,2] oxaphosphorin,6-[(1-oxido-2,6,7-trioxa-1-phosphabicyclo [2.2.2] oct-4-yl) methoxy]-, 6-oxide (DOPO-DP) and melamine (Mel) (Figure 8):

(b) A DOPO derivative phosphorus (P) flame-retardant, i.e., 3-(6-oxidodibenzo [c, e] [1,2] oxaphosphinin-6-yl) propenamide (DOPO-DA) and melamine (Figure 9):

The flammability was determined through UL 94-VB vertical burning tests (EN 60695-11-10). This very simple and effective test is shortly described in the following. A specimen is held in the vertical position. A burner flame is applied to the free end of the specimen for two conventional time intervals (10 s) separated by the time it takes for flaming combustion to cease after the first application. The flammability evaluation is based on the duration of flaming and glowing combustion after burner flame applications, whether or not cotton placed below the specimen is ignited by flaming drips, and whether or not the flame finally reaches the holding clamp.

Self-extinction fire behavior (UL 94-V0 rating) was obtained when adding proper mixtures of DOPO-DP or DOPO-AD and melamine [25,26]. In the second case, a P content equal to 2% was enough. It is a very interesting result if one takes into account the difficulties with the flame retardance of epoxies cured with more eco-friendly non-aromatic hardeners. In both cases, the fire mechanisms were studied with the aid of cone calorimetry, pyrolysis combustion flow calorimeter (PCFC), direct inlet probe mass spectroscopy (DIP-MS), pyrolysis-gas chromatography mass spectrometry (PY-GC-MS) and char analysis through attenuated total reflectance Fourier transform infrared (ATR-FTIR) and energy dispersive X-ray spectroscopy (EDX). It was proven that the in situ-formed nanosilica plays a relevant synergistic role with traditional flame retardants. In particular, melt dripping suppression is strictly related to the presence of silica nanoparticles. The nanosilica formed with the “chronological two-step” process allowed us to also have a good distribution of humic acids (HAs), an abundant biowaste, and to exploit its flame-retardant properties [29]. The addition of HAs alone prevented melt dripping. UL 94-V0 classification (self-extinguishing behavior) was reached at 6% HAs and only 1% P content obtained by the addition of the very popular flame-retardant ammonium polyphosphate (APP). This, of course, also represents an interesting example of waste-to-wealth transformation.

## 5. Insights into the In Situ Nanocomposite Structure and Formation through HRTEM and SAXS

**Figure 10:**
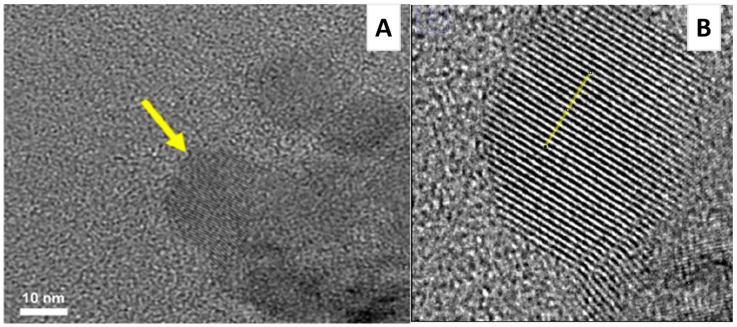
(**A**) HRTEM micrograph of EPO_6%Si_1.25, (**B**) zoom of A, showing how the sheet thickness was evaluated. Reprinted with permission from [31]. Copyright 2023 American Chemical Society.

**Figure 11:**
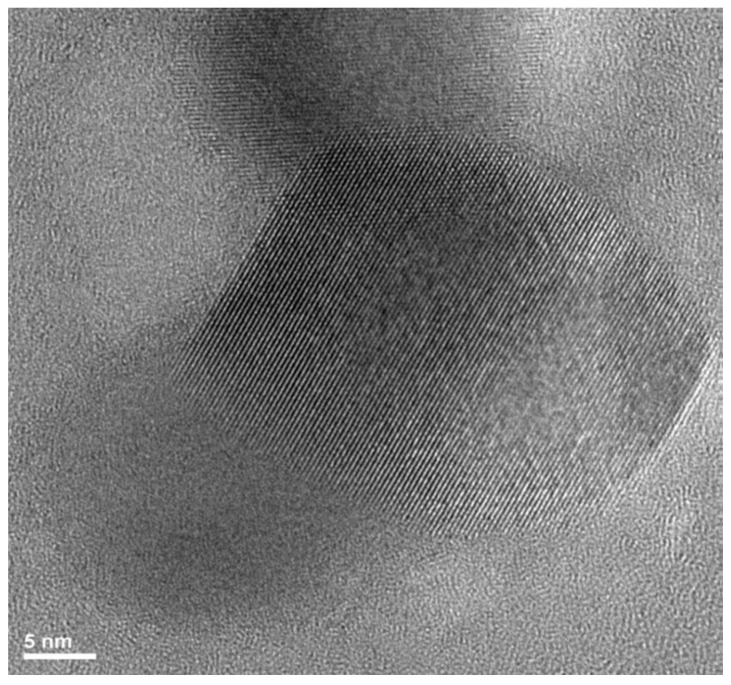
HRTEM micrograph of EPO_6%_Si_2.32. Reprinted with permission from [32]. Copyright 2023 American Chemical Society.

Surprising results were, very recently, obtained when submitting epoxy silica nanocomposites formed through the “chronological two-step” process to HRTEM and SAXS (Figure 10 and Figure 11) [31,32].

The two micrographs refer to epoxy/silica nanocomposites of silica content 6% differing for the weight ratio of the two inorganic precursors (TEOS/APTES): 1.25 and 2.32, respectively. The HRTEM micrographs of EPO_6%Si_1.25 and EPO_6%Si_2.32, [31,32] show a surprising result. The very fine nanoparticles have a multisheet structure instead of the gel one expected when a sol–gel process is applied. The sheet thickness evaluated, indicated in Figure 10, is 0.34 nm. The particles’ sizes were estimated to be about 5–9 nm in good accordance with the values obtained through SAXS investigation [31]. Even the sheet thickness, 0.34 nm, is quite close to the lower typical size value assessed by SAXS (0.457 nm). Furthermore, the HRTEM results are also in good agreement with previously reported NMR results. In fact, when submitting to Si and C CPMAS NMR, both spectra agreed on the full involvement of silicon atoms in the condensation reactions. Similar results were also reported by Chiang et al. [71]. This is quite surprising; usually, non-condensed or also non-fully hydrolyzed moieties are found through NMR [2,14,21]. The final structure, in fact, is the result of the simultaneity of the condensation and hydrolysis reactions (Reactions (2)–(4)), leading to the formation of a gel; the formation of a “spanning gel” prevents further condensation.

**Figure 12:**
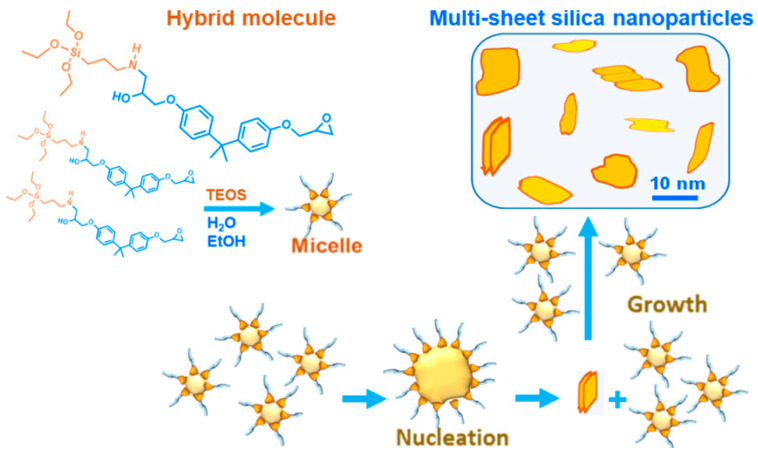
Scheme of the formation mechanism of multisheet silica nanoparticles in the epoxy matrix. Reprinted with permission from [31]. Copyright 2023 American Chemical Society.

A mechanism [31,32] that could explain the experimental results was proposed. Hybrid molecules (silanized epoxy species) would form in the first step of the “chronological two-step” process from APTES and DGEBA, as described in the mechanism represented in Figure 12. Upon the addition of water and ethanol, micelle-like nanodroplets might be produced (through hydrolysis and polycondensation reactions), where TEOS, initially dissolved in the epoxy resin, migrates forming oligomeric species. The hybrid molecule in fact has at one end a strong affinity with epoxy and, upon hydrolysis of the ethoxy groups, shows strong polar groups at the other end. It may, therefore, play the role of a surfactant molecule, allowing the formation of the nanoreactors for the reactions of TEOS.

Nuclei of the new multisheet silica nanoparticles would form as represented in the scheme. Nucleation would involve the aggregation of the micelles till a critical size at which disaggregation would result in very fine nanoparticles (the nuclei) and micelles containing the residual water and alcohol. Upon aggregation of the micelles with the already formed nanoparticles, the structural units present therein would be transferred, thus allowing for nanoparticle growth. All this is very similar to the mechanism proposed for the crystallization of inorganic glasses in [87]. Similarly, growth would occur through the addition of “smaller structural units” present in the matrix to the already formed crystal surface. In the present case, these “smaller structural units” would be the oligomeric structures present in the micelles. In this way, according to the HRTEM, SAXS and NMR results, the formation of the ordered multisheet nanoparticles, instead of gel particles, is justified.

When comparing the two micrographs, it can be observed that the effect of changing the precursors’ weight ratio is that larger multisheet silica nanoparticles are obtained when the TEOS/APTES is increased. Taking into account that total silica content is the same, this suggests that more numerous but finer nanoparticles are produced when increasing the APTES content. This is in good agreement with results reported in the literature. Adnan et al. [78] reported that “reducing the amount of coupling agent resulted in an increase in the cluster size (~110 nm) and the free-space length (205 nm)”; from a mechanism perspective, this finds an explanation in the different effects APTES concentration is expected to have on nucleation and particle growth rates [32]. Evidences of the formation of co-continuous phases already hypothesized in the past by many authors were found through DMA [63,64,65,66,67,68,81,82,83]. In this regard, it is worth observing that, according to the mechanism, two kinds of nanoparticles would be present when the hardener is added: multisheet silica nanoparticles and residual micelles. The co-continuous structure could well be the result of epoxy polymerization around the two different nanometric phases [32]. Also, the glass transition plot vs. APTES/epoxy weight ratio could find an explanation [32]. The mechanism also led to interesting perspectives. In fact, on its basis, the formation of nanoparticles of a different chemical nature other than silica in epoxy (or other hydrophobic resins) can be foreseen [33].

We would, however, need a nanoparticle precursor satisfying the following two conditions:

- Solubility in the hydrophobic resin;
- The ability to result in the new phase through hydrolysis and, possibly, polycondensation.

Mg(OC_2_H_5_)_2_ is a Mg(OH)_2_ precursor that simply fulfills the above requests. Epoxy nanocomposites containing Mg(OH)_2_ nanocrystals were successfully produced by simply substituting Mg(OC_2_H_5_)_2_ to TEOS in the “chronological two-step” process, as proved through XRD, HRTEM and TGA [33].

## 6. Conclusions

The sol–gel chemistry proves to be a valuable route for the synthesis of silica nanoparticles and silica/epoxy nanocomposites with enhanced properties, with respect to the neat epoxy ones.

Well-assessed procedures allow us to prepare monodispersed silica nanoparticles, exhibiting easily tunable sizes and possibly mesoporous structures, as well as possessing very high specific surfaces. The nanoparticles’ surface can be easily modified through the use of a silane coupling agent in order to achieve a fine dispersion in the polymeric matrix or even tailor the interface in the prospect of the applications. Moreover, they can be the core of highly sophisticated and complex nanoparticles specifically designed for a desired application, exploiting the basic sol–gel reaction scheme. This is well-proven by the list, which is of course not exhaustive, of nanocomposites obtained by the dispersion of these nanoparticles in epoxy (ex situ procedure) reported in this paper.

A particular focus was placed on the synthesis of nanocomposites based on in situ-generated silica nanoparticles. In situ processes may be, in fact, designed in such a way that the involved solvent is significantly reduced, while the time- and money-consuming steps of the ex situ method are overcome. Improvements of thermal stability, mechanical properties, glass transition temperature, storage modulus, barrier properties and a better high voltage insulation with respect to the neat epoxy ones were reported.

More recently, important modifications to the procedures were applied with batch compositions, including reagents other than those strictly necessary to generate the nanosilica. The addition of ionic liquids allowed us to produce rubbery nanocomposites possessing an up to 6-times higher modulus and tensile strength, as well as a more than 10-times higher energy to break than unmodified silica/epoxy. It was also demonstrated that flame retardants can be easily introduced, allowing the nanocomposite to reach, at very low P contents, a UL 94-V0 rating corresponding to self-extinguishing fire behavior thanks to a synergistic action with nanosilica. Also, humic acids, a known and abundant biowaste, could be introduced as flame retardant, assuring, in formulations with other traditional ones, UL94-V0 rating, as a good example of a waste-to-wealth approach. On the one hand, all this paves the way to new perspectives of applications. In fact, very often, the satisfaction of severe fire safety regulations is a “sine qua non” prerequisite for the use. On the other hand, this proves that the in situ strategies may proceed beyond the traditional reagents’ batch compositions, comprising strictly polymeric and nanosilica precursors.

Finally, very recently, the use of HRTEM and SAXS proved that, at least in the case of the “chronological two-step” procedure, the in situ-generated nanosilica exhibits a surprisingly ordered multisheet structure. A mechanism, borrowed from the classical theory of crystallization of inorganic glasses, has been proposed that allows us to provide an explanation for the NMR, FTIR and DMA results collected on the same system as well. A new interpretation of the origin of the bicontinuous-phase structure reported by many authors is also given. It is worth pointing out that the mechanism foresees the possibility to synthesize nanocomposites made of different natures, both matrix and filler, and defines the characteristics of the precursor. The synthesis of a nanocomposite containing Mg(OH)_2_ nanocrystals seems to support these expectations.

In conclusion, the ex situ strategy allows us to produce very complex nanoparticles to disperse into polymeric matrices. The in situ strategy is a very promising synthesis strategy that allows us to produce in an easy and eco-friendly manner nanocomposites (not only epoxy/silica) for outstanding applications. The mechanism of nanoparticles formation could also help to design synthesis strategies of nanocomposites with in situ-generated nanoparticles more complex than the ones obtained, until today, with this strategy.

## Data Availability

The data presented in this study are available on request from the corresponding author.
